# Development of Self-Healing Polyurethane and Applications in Flexible Electronic Devices: A Review

**DOI:** 10.3390/polym17172274

**Published:** 2025-08-22

**Authors:** Jie Du, Xinlan Zhao, Yang Li, Wanqing Lei, Xing Zhou

**Affiliations:** 1 School of Art and Design, Xi’an University of Technology, Xi’an 710048, China; 2 Faculty of Printing, Packaging Engineering and Digital Media Technology, Xi’an University of Technology, Xi’an 710048, China

**Keywords:** polyurethane, self-healing, flexible electric device

## Abstract

Traditional polyurethanes have gained widespread application due to their excellent mechanical properties, wear resistance, and processability. However, these materials are susceptible to cracking or fracture under environmental stresses. In recent years, self-healing polyurethanes have garnered significant attention as a critical research field owing to their key capabilities, such as repairing physical damage, restoring mechanical strength, structural adaptability, and cost-effective manufacturing. This review systematically examines the healing mechanisms, structural characteristics, and performance metrics of self-healing polyurethanes, with in-depth analysis of their repair efficacy across various applications—particularly in flexible electronic devices. It demonstrates that self-healing polyurethanes overcome traditional failure modes in flexible electronics through self-repair-function integration mechanisms. Their stimuli-responsive healing behavior is driving the evolution of this field toward an intelligent regenerative electronics paradigm.

## 1. Introduction

Polymers exhibit deficiencies in terms of mechanical properties and heat resistance when compared to metallic and inorganic non-metallic materials. This can result in damage to the polymer surface, manifesting as surface scratches and cracks, due to external forces. The accumulation of such damage can lead to a decline in the performance and service life of materials, which are then discarded as industrial waste, thereby exerting an environmental impact. Conventional repair methodologies employ thermoplastic polymers as a binding agent to repair cracks caused by damage to organic polymers through the application of heat, high temperature, or high pressure. This process can enhance the strength of the damaged area on a macro level. However, the presence of small cracks in organic polymers is not conducive to this process. The utilization of organic polymers, endowed with the capacity for self-healing, has been demonstrated to surpass the limitations inherent in conventional healing methodologies. In instances where a polymer possessing such self-healing properties sustains damage or minor fissures, it undergoes autonomous healing without the necessity for additional binders, thereby mitigating the risk of further damage.

Self-healing materials are defined as those that possess the capacity to repair their own physical damage, in addition to restoring their physical properties [1]. The extension of the service life of the material under consideration is conducive to a reduction in material waste, thus engendering an increase in material sustainability. In recent decades, there has been a rapid development of self-healing composites of metals [2,3], ceramics [4], and polymers. According to the Fortune Business Insights report, the global self-healing materials market reached USD 5.58 billion in 2023, with polymer-based materials dominating the sector. In this context, polymers have emerged as a primary research focus for self-healing materials due to their distinctive characteristics, including superior mechanical properties, effective structural adaptability, and minimal fabrication cost [5,6,7,8].

Waterborne polyurethanes (WPUs) have gained significant popularity in numerous fields due to their environmentally friendly characteristics. These materials possess a number of notable properties, including good abrasion resistance, flexibility, hardness, impact resistance, gloss, chemical resistance, low flammability, durability, high adhesive strength, ease of cleaning, low viscosity, and weathering resistance [9,10]. WPUs are thus of significant industrial importance, with applications including coatings, adhesives, ink binders, glass fibers, paper sizing, synthetic leather, biomaterials, and waterproof textiles. Within the domain of packaging, WPU can be utilized in a variety of applications, including its role as a printing ink linker, as well as in the production of packaging film and coated film. In particular, polyurethane solutions have been shown to exhibit a certain degree of adhesion to substrates such as plastics, aluminum foils, and paper, among others. Consequently, WPU is extensively employed as a coating for flexible packaging printing, where it is applied to the surface of packaging materials to create a polyurethane protective coating. This coating exhibits exceptional abrasion and oil resistance properties, making it a suitable choice for various applications in the field of flexible packaging. During the preparation of waterborne polyurethane, foam formation is inevitable due to mechanical agitation during emulsification and the presence of surfactant. While an appropriate amount of foam contributes to emulsion stabilization and film formation, excessive foam can lead to coating defects such as craters [2]. Therefore, control measures such as adding defoamers or vacuum degassing are required.

However, WPUs are easily damaged by external conditions, resulting in cracks or even fractures, which seriously affects the various properties of waterborne polyurethanes. In dry environments, rapid moisture evaporation can induce crack formation, while in humid conditions, cyclic moisture absorption/swelling followed by drying/shrinkage leads to fatigue cracking. Furthermore, elevated temperatures accelerate both hydrolytic and oxidative degradation of polymer chains. Additionally, prolonged mechanical stress or UV exposure may cause chain breaking, resulting in crack initiation [5,7]. In recent years, many researchers have explored self-healing WPU to achieve the purpose of prolonging the service life, and at the same time, self-healing WPU has ushered in many challenges. For example, the applicability of healing conditions and the conflict between healing and mechanical properties. The relatively low bond energy of disulfide bonds compared to other dynamic bonds shows the advantage of mild healing conditions. However, the compounds commonly used to provide disulfide bonds are bis(4-hydroxyphenyl) disulfide, bis(4-aminophenyl) disulfide, bis(2-hydroxyphenyl) disulfide, diphenyl disulfide, etc., and the high cost as well as the hazardous nature of these monomers greatly limit the application of self-healing WPU [8,9].

The development of self-healing WPU holds significant implications for traditional polyurethane coatings, particularly in intelligent research fields such as flexible sensors and electronic skins under room temperature self-healing property [10]. Incorporating self-healing properties into flexible electronic devices has been demonstrated as an effective strategy to mitigate mechanical damage during operation. This approach effectively extends the service life of materials and devices while reducing material waste, labor replacement costs, and lifecycle expenses, thereby establishing a more sustainable pathway for low-carbon recycling [8].

This review systematically examines the theoretical foundations, preparation methodologies, and application domains of self-healing polyurethanes, with particular emphasis on the regulatory mechanisms and influencing factors of their self-healing properties. A comprehensive analysis is provided regarding the fabrication processes and performance characteristics of diverse self-healing polyurethanes integrated with various external stimuli. The findings conclusively demonstrate that self-healing polyurethanes exhibit substantial application potential in flexible electronic devices, indicating promising prospects for future intelligent materials and electronic systems.

## 2. Self-Healing Polyurethane

Self-healing polyurethane is an intelligent polymeric material that achieves autonomous repair of damage through dynamic covalent bonds or reversible non-covalent interactions, effectively restoring most of its original mechanical properties for three cycling times [10]. In accordance with the principle of self-healing, the categories of self-healing can be divided into two distinct types: exogenous self-healing and intrinsic self-healing [11].

### 2.1. External Self-Healing Polyurethane Material

The externally supported self-healing materials are achieved by pre-embedding micro-containers containing a healing agent and a corresponding catalyst in a matrix [12,13,14,15]. In the event of material damage, the microcontainer that encapsulates the healing agent is ruptured beneath the surface of the material. This results in the release of the healing agent, which reaches the crack created by the damage and reacts with the catalyst within the matrix to repair the crack. This healing mechanism is ordinarily observed to occur at ambient temperature. It has been demonstrated that external conditions, such as light and heat, can accelerate the healing process and enhance the effectiveness of the material. The classification of externally supported self-healing materials is typically based on the structural characteristics of the microcontainer, including mainly two broad categories: microencapsulated self-healing materials and hollow fiber self-healing materials.

#### 2.1.1. Microencapsulated Self-Healing Materials

**Figure 1:**
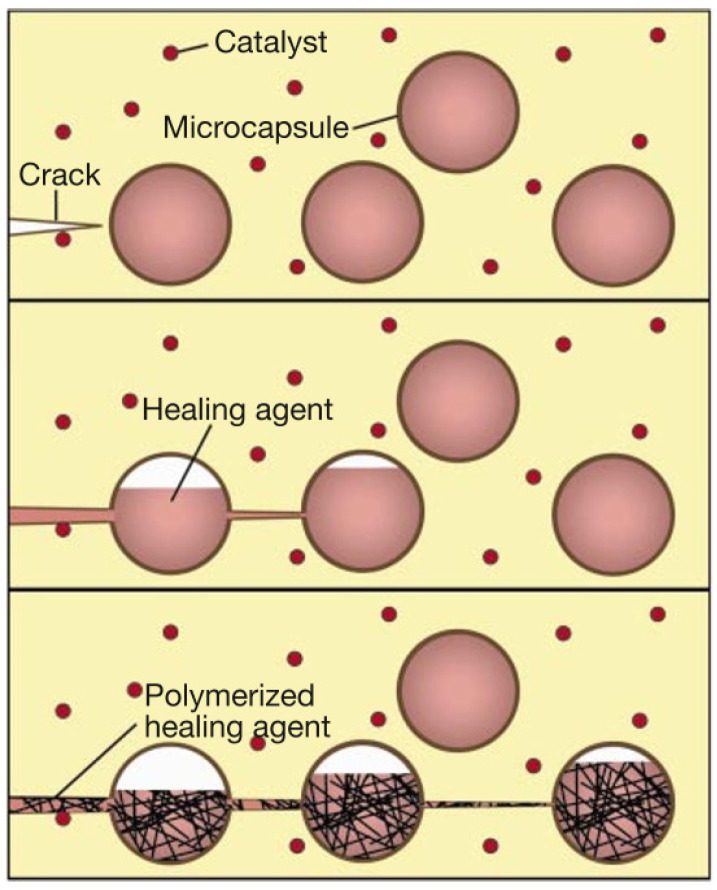
Concept of autonomous healing of microcapsules [16].

White et al. [16] first introduced the concept of self-healing of microcapsules, and this healing mechanism is shown in Figure 1. This system achieves self-healing through ROMP (ring-opening metathesis polymerization), which meets critical requirements, including long shelf life, low monomer viscosity and volatility, and minimal shrinkage during polymerization. The ROMP reaction employs transition metal catalysts (Grubbs’ catalyst) that exhibit high conversion activity while maintaining tolerance to various functional groups, oxygen, and water. This reaction can polymerize dicyclopentadiene (DCPD) at room temperature within minutes, forming a tough, highly cross-linked polymer network. When cracks form in the epoxy matrix, the microcapsules rupture to release the healing agent, which then penetrates to the crack surface via capillary action. Upon contact with the embedded catalyst in the matrix, the DCPD polymerizes within minutes to complete crack repair. The microcapsules with urea-formaldehyde shells (50–200 μm) containing DCPD were prepared using standard microencapsulation techniques. The microcapsule shells provide a protective barrier between the catalyst and DCPD to prevent premature polymerization during composite fabrication. This pioneering system established a fundamental framework for subsequent self-healing research.

The research prospect of microencapsulated self-healing is promising, primarily due to its straightforward repair method and notably high repair efficiency. However, a compatibility issue has been identified between the microcapsules and the substrate, with the embedding of microcapsules having a detrimental effect on the substrate’s original structure, consequently altering the material’s mechanical properties. Furthermore, following the occurrence of microcapsule and polymerization, the microcapsule is rendered incapable of initiating the healing reaction. This phenomenon causes a limited number of healing times for the material and the inability to satisfy multiple healings in the same place. Consequently, the subsequent microcapsule self-healing system will prioritize the selection of microcapsule material and its compatibility with the substrate, along with other pertinent factors.

#### 2.1.2. Hollow Fiber-Based Self-Healing Materials

Microencapsulated self-healing materials present a significant disadvantage in that they can only undergo a single repair at their damaged site. This limitation has led to the proposal of the concept of hollow fiber self-healing. It has been established that it is analogous to the microcapsule self-healing mechanism. The triggering of both mechanisms occurs as a result of cracks in the microcontainer rupture, the overflow of the healing agent, and the catalyst within the matrix in contact with the catalyst. The catalyst is responsible for triggering the polymerization reaction, thereby achieving the healing of the material. The primary distinction between the hollow fiber type and the microcapsule type pertains to the configuration of the microcontainer. In the hollow fiber type, the healing agent is injected into the hollow fiber, which is prepared in advance. Subsequently, the hollow fiber is composited with the matrix post-treatment [17].

The concept of hollow fiber-based self-healing materials was initially introduced by Dry’s group [18], who proposed their storage within glass fiber tubes. The findings of the impact experiments demonstrated that the repair adhesive exhibited the capacity to migrate along the crack, in addition to facilitating the process of complete healing. Quantitative bending experiments demonstrated that this repair adhesive was also capable of hindering crack expansion. In accordance with this theory, researchers have constructed hollow tubes to meet specific requirements and employed various repair agents to facilitate healing. Jody et al. [19] developed a novel fiber-reinforced plastic utilizing a biomimetic approach which employed a biomimetic approach, to undertake self-repair and visual enhancement of impact damage by a bleeding action from filled hollow fibers.

**Figure 2:**
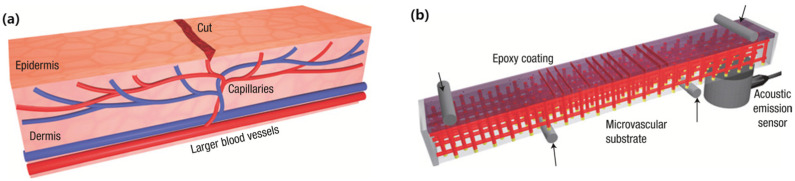
Schematic diagram of 3D microvascular system: (**a**) simulation diagram of capillary network; (**b**) schematic diagram of hollow fiber type self-healing structure [20].

The results of flexural testing have shown that for the lay-up investigated, a significant fraction of flexural strength lost after impact damage can be restored by the self-repairing effect of a healing resin stored within hollow fibers. Toohey et al. [20] have developed a self-healing material based on a three-dimensional microvessel system inspired by bionics and modelled on the human capillary network (Figure 2). The microvascular substrate was fabricated using a robotic deposition system (Model JL2000, Robocasting Enterprises, Albuquerque, NM, USA) via a layer-by-layer approach with fugitive organic ink, forming channels with a diameter of 200 μm. The microvascular matrix was then combined with a brittle epoxy coating containing embedded catalysts to construct a three-dimensional microvascular network capable of delivering healing agents (DCPD healing agent) to cracks in the polymer coating. Experimental results demonstrated that microvascular networks with high catalyst concentration (10 wt%) exhibited healing over multiple cycles, with healing capability ceasing after seven cycles.

The unique structure of hollow fibers necessitates the infliction of greater damage to the matrix before the fibers can be stimulated to rupture once more, thereby facilitating the efflux of the healing agent for self-healing. Concurrently, the intricate configuration of hollow fibers and the substantial expense associated with their fabrication impede the implementation of this form of self-healing, thereby hindering the attainment of large-scale industrial production.

In a word, the hollow fiber self-healing system is suitable for large-scale repairs and can be triggered multiple times, while the microcapsule self-healing system is ideal for rapid localized repair of micro-damage. The two can be combined to achieve multi-scale collaborative self-healing.

### 2.2. Intrinsic Self-Polyurethane Healing Materials

Intrinsic healing is defined as self-healing that occurs as a result of the fracture-reorganization of special chemical bonds contained within the material, thus negating the need for additional healing agents and corresponding catalysts. The classification of such special chemical bonds is typically performed into two categories [21,22]. Dynamic non-covalent bonds, which include metal-ligand bonding, ionic bonding, hydrogen bonding, π-π stacking, and host-guest interactions. Dynamic covalent bonds, which encompass Diels–Alder bonding, acylhydrazone bonding, disulfide bonding, diselenide bonding, and others. Intrinsic self-healing materials have attracted considerable attention due to the tunability of their material structure.

#### 2.2.1. Dynamic Non-Covalent Bonds

(1) Metal coordination bond

The metal-ligand bond, the bonding energy of which falls between the van der Waals forces and traditional covalent bonds, is defined as a supramolecular force. This is formed by the complexation reaction between a metal cation (e.g., Cu^2+^, Ca^2+^, Mn^4+^, Fe^2+^, Mg^2+^, Co^2+^, Zn^2+^, etc.) and an organic ligand. The complexation reaction is able to be stimulated by certain external conditions. Reversible fracture-reorganization can occur due to the relatively good localization of the ligand bond, meaning it can be flexible in its choice of the appropriate ligand point [23]. This has wide-ranging applications in self-healing materials.

**Figure 3:**
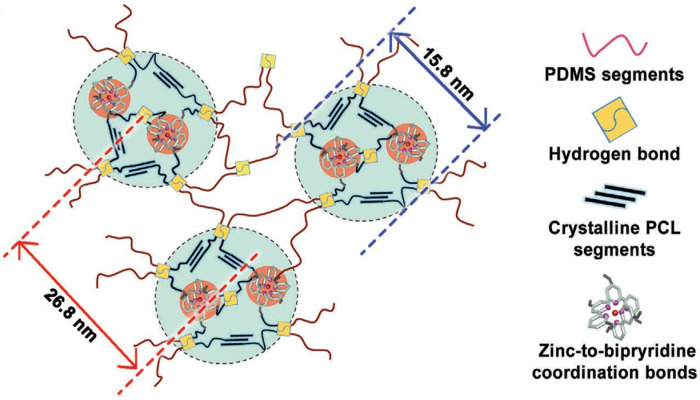
Schematic diagram of the self-healing polyurethane elastomer structure [24].

Wang et al. [24] designed a block polymer and prepared polyurethane elastomers with recyclable and self-healing properties (Figure 3) by dynamic layered structure design. The formation of hydrogen bonds within the polyurethane, in conjunction with metal-ligand bonding facilitated by bipyridine and Zn^2+^, resulted in the emergence of a microphase-separated structure. This structural modification has been demonstrated to enhance the mechanical properties of the polyurethane. The self-healing property of the polymer material, when subjected to metal-ligand bonding, renders the polymer recyclable. Guan Zhibin et al. [25] developed a microphase separation technique for the synthesis of supramolecular materials, characterized by exceptional mechanical properties and intrinsic self-healing capabilities. The introduction of polystyrene to the hard part and imidazole to the soft part of the material is of particular interest. On the one hand, polystyrene has been shown to improve the mechanical properties of the material. On the other hand, the formation of a metal-ligand bond through imidazole-Zn^2+^ endows this supramolecular material with self-healing properties. The supramolecular material was shown to possess the capacity for autonomous regeneration at ambient temperature for a duration of three hours, resulting in the restoration of the Young’s modulus to its original state.

(2) Hydrogen bond

In the presence of electrostatic forces, hydrogen atoms have been observed to be attracted to atoms or groups with higher electronegativity, such as nitrogen, oxygen, or fluorine atoms. Once the pairing occurs, a hydrogen bond is able to form between the two atoms. The bond strength of hydrogen bonds is lower in comparison to covalent bonds, resulting in a faster process of bond breakage and reorganization [26]. The formation of hydrogen bonds can occur in a variety of numbers, and they are categorized as either single or multiple hydrogen bonds. In general, the mechanical properties of polymers with single hydrogen bonds are suboptimal due to the weak physical crosslinking network composed of single hydrogen bonds; this is compensated for by multiple hydrogen bonds [27].

**Figure 4:**
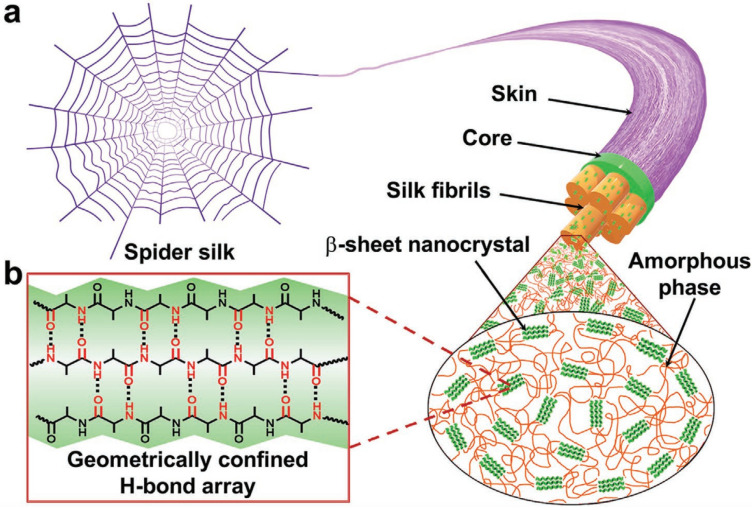
Schematic illustration of the structure of spider silk (**a**) the spider structure; (**b**) the main H-bond array in ordered structure [28].

Inspired by high-strength spider silk, Li et al. [28] selected hexanedihydrazide as a chain extender to fabricate a supramolecular poly(urethane urea) (SupraPU) elastomer. This elastomer exhibits high tensile strength with an ultimate engineering stress of 75.6 MPa and achieves 100% self-healing efficiency after 36 h at 100 °C. When shredded into fragments and subjected to 5 MPa pressure at 130 °C for 0.5 h, it fully recovers its original form, demonstrating exceptional recyclability. These remarkable properties originate from meticulously designed hydrogen bonding segments, comprising ureido-4-pyrimidinone (UPy) and urethane moieties connected by flexible alicyclic six-atom spacers. These segments form geometrically ordered hydrogen bond arrays between flexible polymer chains and aggregates, mimicking the spider silk structure as illustrated in Figure 4.

(3) Ionic bond

Ionic (or electrostatic) interactions are defined as physical bonds formed through electrostatic interactions between two ionic substances with opposite charges. These interactions are characterized as non-saturated and non-directional and result in a fracture-reorganization response to changes in pH, thereby completing the self-healing behavior. The high response speed and high viscosity of polymer materials containing ionic bonds have led to their significant attention in the field of self-healing materials.

**Figure 5:**
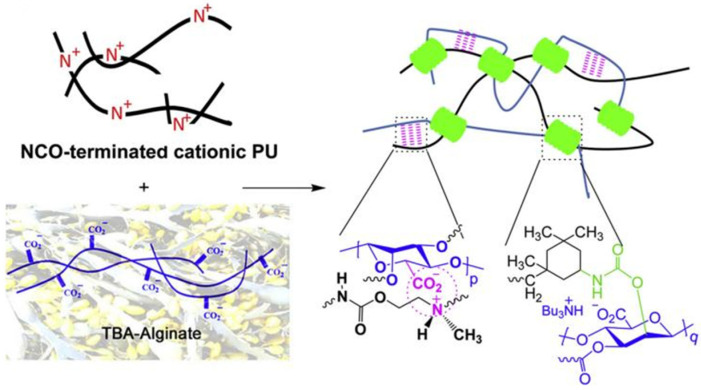
Schematic illustration of dual networks formed by alginate-based supramolecular ionic polyurethanes (ASPUs), green regions represent for the covalent linkages of urethanes between hydroxyl groups of alginate and isocyanates of NCO-terminated prepolymer serve as strong bonds to maintain the shape of material and purple dash lines refer to ionic interactions between carboxylate groups of alginate and tertiary ammonium moieties of cationic PU toughen the materials by bond rupture [29].

Daemi et al. [29] successfully synthesized and characterized an alginate-based polyurethane elastomer exhibiting supramolecular interactions in the solid state. The material was constructed using ionic supramolecular motifs, as depicted in Figure 5. These interactions confer simultaneous high rigidity and toughness to the elastomer, demonstrating broadly tailorable mechanical properties modulated by alginate content—reaching a tensile strength of 48 MPa and Young’s modulus of 93 MPa. The elastomer displays rapid and exceptional self-healing capability, with healing efficiencies of 87.3% in the first cycle and 70.2% by the third healing cycle. Jiang et al. [30] developed a self-healing polyurethane by incorporating tertiary amine groups and carboxyl groups into the polymer matrix to form ionic interactions. The prepared polyurethane exhibited room temperature self-healing capability with an impressive healing efficiency of 104.72%, along with excellent shape memory properties and processability.

(4) π-π stacking

π-π stacking is a non-covalent bonding interaction force between aromatic rings, which is a relatively weak interaction force usually found in electron-rich and electron-deficient aromatic compounds. π-π stacking is widely found in living organisms, such as nucleobase stacking and protein folding [31].

**Figure 6:**
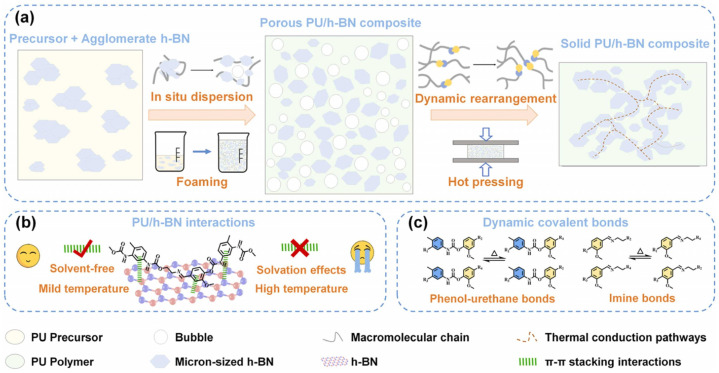
Schematic diagram of the two-step foaming-pressing strategy based on dynamic covalent chemistry. (**a**) The agglomerated micron-sized h-BN are mixed with the PU prepolymer and dispersed by the in-situ generated bubbles during the foaming process, resulting in the immobilization of the dispersed h-BN within the porous polymer; afterward, the porous PU/h-BN composite is hot-pressed and the dynamic reactions of implanted dynamic covalent bonds during this stage allow the network structure to rearrange, generating densified composites with homogeneously dispersed fillers. (**b**) The π-π stacking interactions between PU matrix and h-BN can play a more prominent role under a solvent-free and mild-temperature conditions. (**c**) The implanted phenol-urethane bonds and imine bonds undergo exchange reactions to enable network rearrangement [32].

Wu et al. [32] proposed a mild and solvent-free strategy combining classical chemical foaming with dynamic covalent chemistry to fabricate polyurethane (PU)/hexagonal boron nitride (h-BN) composites. As illustrated in Figure 6, aromatic moieties in the PU matrix derived from toluene diisocyanate (TDI) physically adsorb onto h-BN surfaces through π-stacking interactions. Leveraging the material’s reversible characteristics, the composite achieves a 95% healing efficiency.

Although π-π stacking interactions endow self-healing polyurethane with highly efficient self-repair capabilities, such materials still suffer from inherent limitations, including low mechanical strength, insufficient modulus, and the requirement of external stimuli to trigger the healing process. Consequently, research on developing self-healing PU elastomers relying solely on π-π stacking interactions remains relatively limited. Most current studies focus on constructing synergistic systems that combine π-π stacking with other reversible chemical bonds (such as hydrogen bonds and coordination bonds). By leveraging the structural stability advantages of π-π stacking under environmental variations and incorporating the reinforcement effects of other dynamic bonds, researchers aim to develop intelligent self-healing supramolecular composite materials with enhanced mechanical strength and more stable bonding energy.

Yuan et al. [33] successfully fabricated a PU-10% CNC composite elastomer by uniformly dispersing cellulose nanocrystals (CNCs) in a polyurethane matrix via a solvent exchange method. The material achieves an exceptional 91.8% self-healing efficiency at room temperature through synergistic interactions between multiple hydrogen bond networks formed by hydroxyl groups on CNCs and π-π stacking, while demonstrating a remarkable enhancement in tensile strength from 3.5 MPa to 24.6 MPa. Furthermore, the composite exhibits excellent resilience and complete chemical recyclability, offering novel design principles for developing high-performance, environmentally friendly elastomers.

#### 2.2.2. Dynamic Covalent Bonding

(1) Diels–Alder bond

DA bonds are formed through an addition reaction of an olefin with a conjugated olefin, which is a [4 + 2] cycloaddition reaction. The fabrication of self-healing materials based on DA bonds is achieved by means of temperature control, which results in the breakage and reorganization of DA, thereby imparting self-healing properties to the materials.

Tu et al. [34] developed a three-dimensional network structure using functionalized graphene oxide (FGO) and modified carbon nanotubes (FMCNTs) to establish an excellent conductive pathway within the polymer matrix. By incorporating reversible Diels–Alder (DA) chemical crosslinks into waterborne polyurethane (WPU), they fabricated conductive and self-healing flexible electronic materials (FGO/FMCNTs/WPU) through an infiltration method. Experimental results demonstrated that the composite exhibits outstanding thermomechanical properties, achieving a stress of 9.58 MPa, a strain of 207%, and an electrical conductivity of 0.0283 S/m at a nanomaterial concentration of 6 mg/mL.

**Figure 7:**
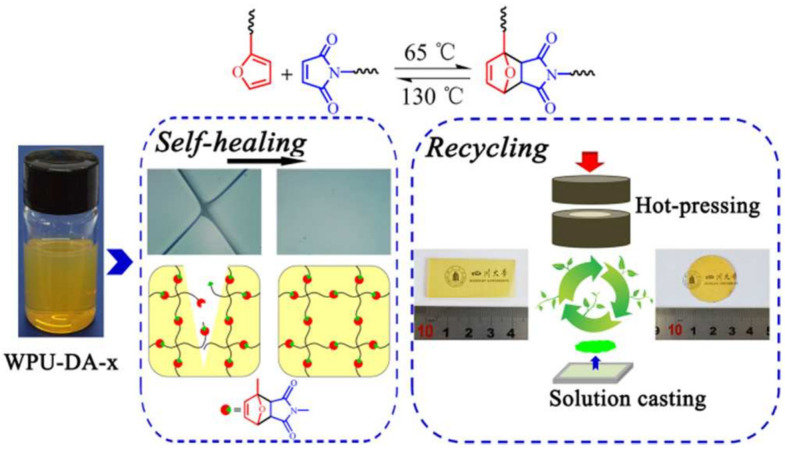
Heat-driven self-repair of recoverable waterborne polyurethane [35].

Fang et al. [35] introduced dynamic amine (DA) bonds into the polymer backbone with a view to preparing a thermally driven, self-healing waterborne polyurethane film (WPU-DA-x) based on the DA fracture-reorganization reaction. This also possesses recyclable properties, as demonstrated in Figure 7. Following a self-healing process at 130 °C for 30 min and subsequently at 65 °C for 24 h, the overall performance of WPU-DA-x was restored, with WPU-DA-6 demonstrating the highest healing rate (92.5%). Concurrently, WPU-DA-x films can be recovered through the utilization of hot pressing and solution casting methodologies. This demonstrates the great potential of WPU-DA-x as a smart material. Du et al. [36] prepared a lignin self-healing polyurethane (PUDA-L) by replacing a portion of polyurethane raw material with lignin, combining lignin with DA bonding and hydrogen bonding. The excellent self-healing ability of PUDA-L originates from the internal DA bonding with the crosslinked hydrogen bonding. Following the application of heat to the PUDA-L specimen at a temperature of 130 °C for a duration of 4 h, the material demonstrated complete healing, exhibiting a self-healing efficiency of 100%. The developed self-healing PUDA-L elastomers have the potential for utilization in sensors and smart skin applications.

(2) Reversible acylhydrazone bond

The reaction of hydrazide with a carbonyl group (aldehyde or ketone) creates a reversible acylhydrazone bond, which breaks and then reforms in response to aniline or acid stimulation. It can therefore be concluded that the addition of aniline or pH adjustment can lead to self-healing of materials containing reversible acylhydrazone bonds.

**Figure 8:**
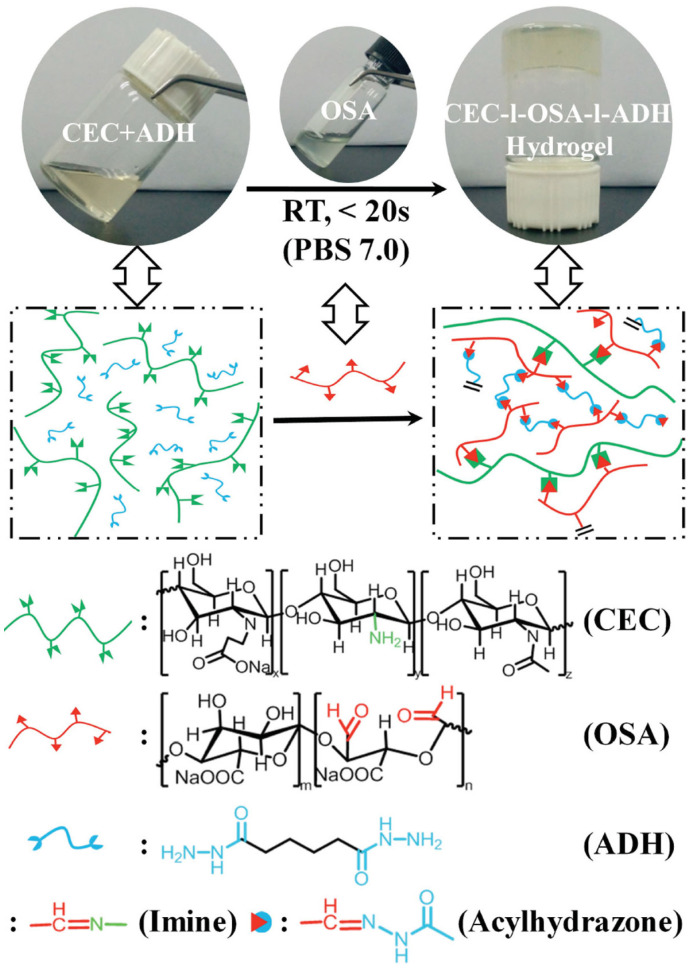
Synthesis protocol of the CEC-1-OSA-1-ADH hydrogel [37].

Zhao et al. [37] developed a novel polysaccharide-based self-repairing hydrogel (CEC-1-OSA-1-ADH), as illustrated in Figure 8. The hydrogel network exhibited dynamic acylhydrazone and imine bonds, enabling dynamic fracture and reorganization to occur under mild conditions. The self-healing capability of CEC-1-OSA-1-ADH hydrogel was substantiated through macroscopic self-healing tests, rheological recovery tests, and analogous methodologies. The hydrogel demonstrated a healing efficiency of up to 95%, a feat accomplished under conditions devoid of external stimulation. Furthermore, the hydrogel exhibits not only effective self-healing properties but also demonstrates excellent cytocompatibility and cell release, characteristics that underscore its considerable potential for biomedical applications.

(3) Imine bond

The imine bond is defined as a dynamic, reversible covalent bond formed by the condensation of an amino group with an aldehyde group, resulting in the formation of a C=N bond. This bond exhibits dynamic properties associated with self-healing and reprocessability without the need for external stimuli. Consequently, materials based on reversible imine bonds have garnered significant interest from the scientific community.

**Figure 9:**
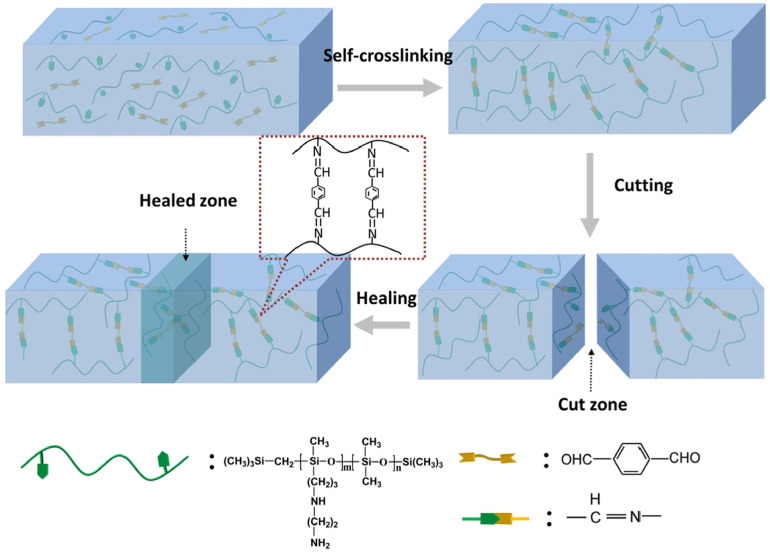
Schematic diagram of HPDMS preparation and self-healing process [38].

Wang et al. [38] successfully prepared healing transparent PDMS elastomers (HPDMS) based on imine bonding using amino-modified polydimethylsiloxane (PDMS) and 1,4-diformylbenzene (DFB) as the raw materials. The formation of imine bonds was demonstrated by infrared spectroscopy and Raman spectroscopy, and the reversible imine bonds could promote the self-healing of fracture surfaces without the stimulation of external conditions. The healing mechanism is shown in Figure 9. The healing rate of HPDMS was found to be 42.6% after 5 min and 97% after 1 h. The elastomer also demonstrated excellent recyclability and processability. Wang et al. [39] also established a dual-network self-healing silicone elastomer (DN elastomer) based on PBS and HPDMS on the basis of HPDMS. The formation of imine and B-O bonds was identified by means of infrared (IR) and Raman spectroscopy. The healing of DN elastomers is based on the synergistic effect of the dynamic reversibility of imine bonds and the fracture recombination of B-O bonds. It has been demonstrated that DN elastomers can heal up to 99% for 1 h at room temperature with almost complete healing. The reprocessed elastomer exhibits a recovery of 85% of its mechanical strength. Furthermore, DN elastomers demonstrate elevated light transmittance within the visible spectrum, thus providing novel concepts for transparent silicone elastomers that are endowed with self-healing properties.

Liu et al. [40] prepared a series of EPCN epoxy polymers based on dynamic imine bonds through a one-pot method, using cost-effective industrial-grade terephthalaldehyde and commercially available bisphenol A diglycidyl ether (E-51) as raw materials with D230 as the curing agent. The rigid aromatic rings of terephthalaldehyde and the flexible molecular chains of D230 endowed the material with tunable thermomechanical properties. Meanwhile, the dynamic imine bonds formed through the Schiff base condensation between amino and aldehyde groups imparted the EPCN epoxy polymers with self-healing capability, reprocessability, and thermally adaptive shape memory behavior. Experimental results demonstrated that when EPCN-4 epoxy polymer was subjected to 150 °C, cracks in the material were completely healed within merely 8 min.

(4) Disulfide bond

Among the numerous dynamic covalent bonds, reversible reactions of disulfide bonds have garnered particular interest. Disulfide bonds are known to exhibit weak bond energies; therefore, they are classified as dynamic covalent bonds. These bonds can undergo a reversible reaction known as fracture recombination, provided that the reaction occurs in the absence of external condition stimuli and at relatively low temperatures (60–90 °C). Consequently, disulfide bonds offer a significant advantage in the synthesis of dynamic polymers [41,42].

**Figure 10:**
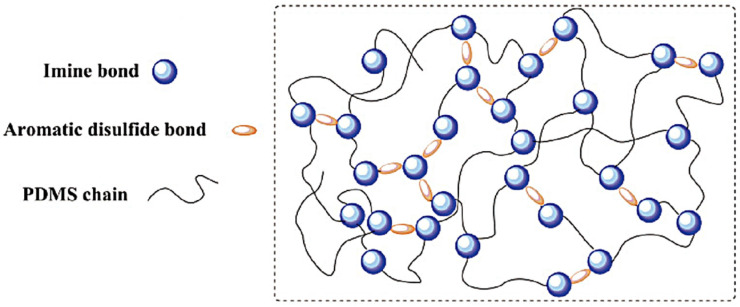
Schematic diagram of the poly(dimethylsiloxane) elastomer structure based on disulfide bonds [45].

In a seminal study, Ye et al. [43] pioneered the development of self-healing aqueous polyurethanes, utilizing a sophisticated combination of PTMG as soft segments, IPDI as hard segments, and 2-hydroxyethyl disulfide (HEDS) as a disulfide bond donor. The zeta potential of these materials was found to be a reliable indicator of their stability, with tensile testing providing a quantitative assessment of their self-healing capacity. The self-healing efficiency of the samples was found to be up to 96.17% after 4 h of heat treatment at 70 °C, and the healing efficiency of the samples was 84.21% after 24 h of healing at room temperature. Conversely, the presence of disulfide bonds in the waterborne polyurethanes rendered them reprocessable. The tensile strength of the samples was measured to be 3.39 MPa (2.85 MPa for the original samples) after 20 min of heat pressing the polyurethane fragments at 130 °C. In the study by Wan et al. [44], a series of self-healing waterborne polyurethanes containing disulfide bonds in the main chain were prepared. The effects of the molar ratios between the introduced HEDS and a hydrophilic chain extender, DMPA, on the self-healing properties of the aqueous polyurethane were investigated. The findings demonstrated that the optimal healing performance of the aqueous polyurethane was attained at a molar ratio of 1:1, with the scratch healing efficiency of the polyurethane surface reaching up to 90.5% following heat treatment at 65 °C for a duration of 10 min. Lv et al. [45] utilized aromatic disulfides to prepare poly(dimethylsiloxane) (PDMS), as illustrated in Figure 10. The completion of the self-healing process of this polymer was observed to require a mere four hours at ambient temperature. However, it was noted that the tensile properties of the polymer were found to be only 0.15 MPa.

While polyurethane materials incorporating disulfide bonds can demonstrate self-healing capabilities, this modification typically compromises their mechanical performance. Such trade-offs between self-heal ability and structural integrity have significantly constrained the practical applications of these materials. Furthermore, the commercial viability of these systems is limited by the high cost of aromatic disulfide monomers and the challenges associated with their industrial-scale synthesis.

In recent years, the rapid advancement of self-healing materials has significantly expanded their applications and heightened their prominence. Intrinsic self-healing polyurethanes, leveraging advantages such as molecular programmability, unlimited repair cycles, and structural homogeneity, have emerged as the core developmental pathway for intelligent regenerative systems such as flexible electronics and soft robotics. Extrinsic systems, however, face constraints due to finite healing agent capacity and interfacial compatibility issues, necessitating targeted improvements through multi-stimuli-responsive microcapsules, dynamic interfacial compatibilizers, and cascade-releasing vascular networks. The field is poised to evolve toward hybrid architectures that synergize dynamic covalent bonds with microvascular containers, ultimately unifying molecular intelligence with scalable manufacturing.

## 3. Self-Healing Polyurethane Materials with Different Stimulus Responses

### 3.1. Heat-Initiated Self-Healing Polyurethane Materials

Thermally initiated self-healing materials are among the most extensively studied materials in this field. The majority of dynamic covalent and dynamic non-covalent bonds present within intrinsic self-healing materials are capable of being thermally initiated [46,47,48,49], with dynamic bond breaking and reorganization occurring under the influence of thermal conditions. The healing efficiency of thermally initiated materials is generally high, but there are some drawbacks, such as the effect of too high a healing temperature on the material’s own structure, the tendency to age the material, and the inability to precisely locate the breakage.

Jong et al. [50] fabricated a self-healing composite based on the Diels–Alder reaction and resistive heating using bismaleimide tetrafuran (2MEP4F) thermoreversible polymers through vacuum-assisted resin transfer molding. Experimental results demonstrated that when heated to 100 °C for 3 h without applied pressure, the samples could recover up to 90% of their strain energy in the absence of fiber fracture. The second healing cycle achieved 86% strain energy recovery relative to the original specimen, showing slightly reduced efficiency compared to the first healing. The developed composite combines multiple healing capabilities with shape memory effects through resistive heating, representing a novel advancement in self-healing composite materials.

Wang et al. [51] fabricated graphene-thermoplastic polyurethane (G-TPU) composite films. Due to graphene’s exceptional infrared light absorption capability, the graphene sheets absorb infrared radiation, inducing internal lattice oscillations that generate heat. This heat is subsequently transferred through the graphene-based thermal conductive network to the TPU matrix, facilitating rapid heating of the composite film. When the temperature of G-TPU approaches the softening point of TPU, the molecular chains in the soft segments undergo thermal motion, allowing the damaged regions to come into contact under gravity and subsequently form a new polymer molecular network. As the temperature decreases, the TPU gradually solidifies, resulting in scratch healing. Experimental results demonstrated that the composite film with a graphene loading of 2 wt% exhibited optimal self-healing performance, achieving healing efficiencies of 99%, 92%, 81%, 72%, and 62% over five self-healing cycles. Additionally, composite films prepared with low-melting-point TPU were found to be more conducive to achieving higher near-infrared (NIR)-triggered self-healing efficiency.

### 3.2. Photoinitiated Self-Healing Polyurethane Materials

Photoinitiated self-healing materials typically utilize light energy to induce or stimulate the reorganization of chemical bonds at the location of the breakage, thereby facilitating the self-healing of the damaged surface. Light energy is abundant, environmentally friendly, and safe; thus, photoinitiated self-healing materials have attracted significant attention. The field of photo-induced self-healing can be broadly categorized into three distinct areas: photocrosslinking reaction self-healing [52,53,54], photo-replacement reaction self-healing [55,56,57,58,59], and photothermal conversion self-healing.

Photocrosslinking and photoreplacement reactions have analogous healing mechanisms, both of which involve the dynamic breaking and reorganization of chemical bonds at the damaged site induced by light. In contrast, photothermal conversion reactions differ in that light energy is converted into heat energy and the damaged site is locally heated to stimulate self-healing of the polymer. In comparison with the three aforementioned options, photothermal conversion reaction is less complicated to prepare and is utilized extensively. Common nanoparticles with photothermal conversion ability include polypyrrole [60], carbon nanotubes [61,62], graphene oxide [63,64], MXene [65], gold nanoparticles [66,67], silver nanowires [68,69], carbon black [70,71], and polydopamine nanoparticles [72,73,74]. It is evident that both the content and the intensity of light irradiation are pivotal in determining the photothermal conversion ability of nanoparticles.

**Figure 11:**
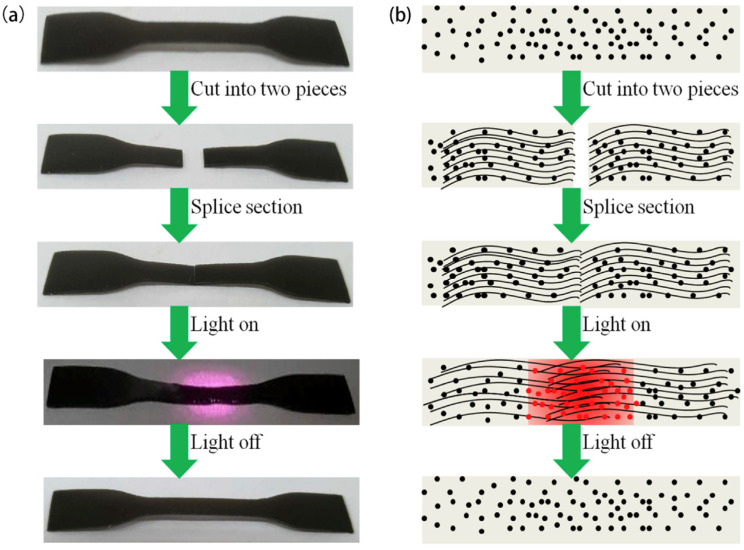
The process (**a**) and mechanism (**b**) of NIR light-induced self-healing [60].

Wu et al. [60] successfully prepared self-healing TPU/PPy nanocomposites that can be induced in the near-infrared (NIR) light by the solution blending method. Experimental evidence has demonstrated that the incorporation of 0.25 wt.% PPy has the potential to enhance the stress-strain characteristics of TPU, with an observed increase from 8.20 MPa and 1540% to 13.50 MPa and 1650%, respectively. Concurrently, the incorporation of PPy has been demonstrated to enhance the healing capacity of TPU/PPy nanocomposites under near-infrared illumination. A mere 30 s is sufficient to restore over 80% of the mechanical properties, as illustrated in Figure 11a. The reinforcing effect of PPy in polyurethane and the healing performance of composite materials serve to mitigate the disparity between the mechanical properties of self-healing materials and their healing efficiency, thereby expanding the scope of applications for PPy.

Li et al. [62] prepared polyurethanes capped with terpyridine ligands by in situ polymerization on the surface of multi-walled carbon nanotubes (CNTs). These were also dynamically crosslinked with Zn^2+^ to obtain metallic supramolecular polymers. The polymer composites demonstrated the capacity for autonomous recovery through the process of self-healing when exposed to near-infrared light (4.2 mW mm^−2^) for a duration of either 30 min or 1 h at a temperature of 90 °C. This metal-supramolecular polymer nanocomposite boasts an array of advantageous properties, including exceptional mechanical strength and innate self-healing capabilities. The material’s vast array of potential applications encompasses structural components, microelectronics, sports equipment, and even artificial skin.

As posited by Lin et al. [75], reduced graphene oxide (r-mGO) was incorporated into polyurethane with a view to preparing NIR photoinduced self-healing nanocomposites based on dynamic DA bonding reactions. The photothermal conversion properties of r-mGO have the potential to impart photoinitiated self-healing properties to the material. It has been demonstrated that the polyurethane can be irradiated by 808 nm near-infrared light at a content of 0.1% r-mGO and that the surface can be increased from room temperature to 100 °C in 1 min. Furthermore, the self-healing of surface damage can be accomplished with a healing efficiency of up to 90%. This near-infrared light has been shown to stimulate self-healing in a manner that enables precise repair of damaged areas while sparing undamaged regions from adverse effects. In certain applications, this self-healing property has the potential to prolong the lifespan of electronic devices, thus providing a new paradigm for the design of next-generation self-healing electronic devices.

### 3.3. pH-Initiated Self-Healing Polyurethane Materials

pH-induced self-healing materials are materials that have the capacity to promote self-healing by altering the acidity or alkalinity of the polymer. Yang et al. [76] utilized whey acid to modify chitosan and composite with 2,6-diaminopurine to prepare a self-healing supramolecular hydrogel (OACS-DAP), which exhibits the dual-healing properties of thermal and pH healing. At pH values lower than 4, a stable hydrogen bonding network can be formed in the hydrogel system, resulting in the hydrogel appearing as a solid. Conversely, at pH values greater than 4, the OACS in the hydrogel and the hydrogen in the DAP undergo a deprotonation reaction, leading to the disruption of the hydrogen bonding network and the transformation of the hydrogel into a liquid or semi-liquid state. The OACS-DAP hydrogel has been demonstrated to possess the capacity for autonomous repair, a process that is facilitated by the hydrogel’s sensitivity to pH.

Mirmohseni et al. [77] investigated the effects of high-load silica capsules containing corrosion inhibitors on the thermal degradation and self-healing properties of waterborne polyurethane (WPU). The silica capsules were synthesized using an oil-in-water (O/W) microemulsion method, while the WPU was prepared via a prepolymer approach. When coating rupture occurs or water penetrates the coating, leading to corrosion on substrates such as iron or aluminum protected by organic coatings, localized pH changes trigger the pH-responsive silica capsules to release 2-mercaptobenzothiazole (MBT) as a corrosion inhibitor, thereby preventing substrate corrosion. The assembled capsules demonstrated pH-dependent release of their core contents.

### 3.4. Electrically Initiated Self-Healing Materials

Electrically triggered self-healing materials refer to the self-healing effect achieved by using the Joule heat generated by an electric current to drive the dynamic bonding within the material to undergo reorganization.

**Figure 12:**
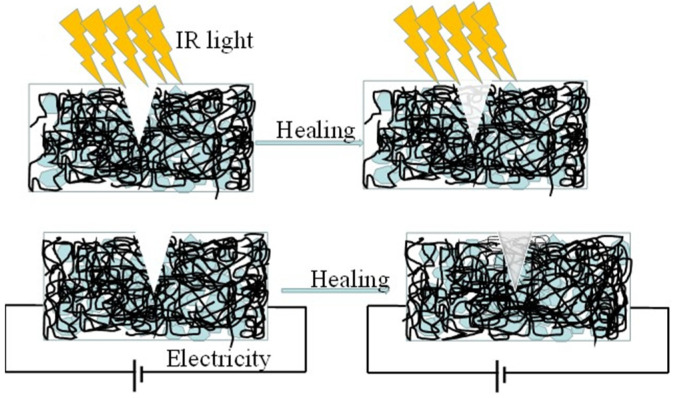
Schematic diagram of self-healing of the composite film by IR light and electricity, respectively [78].

Wang et al. [78] prepared a graphene-thermoplastic polyurethane (G-TPU) flexible conductive film using a blending method, which can stimulate the healing of the film by electrical and infrared light triggering. The excellent infrared absorption and energy conversion/transfer capabilities of graphene allow the temperature of the G-TPU composite film to rapidly rise close to its softening point, thereby enabling self-healing of the composite film through wetting, diffusion, rearrangement, and crosslinking of TPU chains followed by solidification. In the electrically stimulated healing process of the composite film, the temperature also increases due to electrical energy being converted into Joule heating, thus promoting the self-healing behavior of the composite film (Figure 12). Furthermore, it was demonstrated that scratches on the surface of the conductive film could be completely healed at room temperature within 70 s.

### 3.5. Magnetically-Initiated Self-Healing Polyurethane Materials

**Figure 13:**
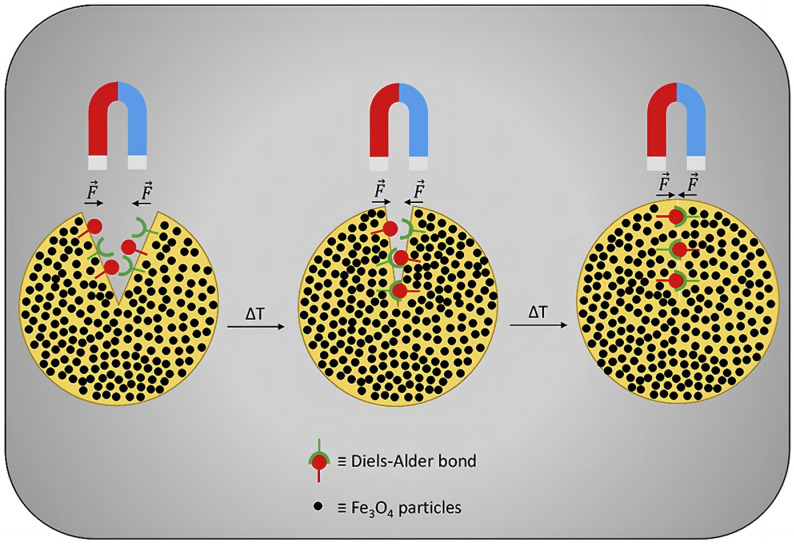
Magnetic healing of magnetite particles and DA bonds [79].

Magnetically initiated materials have been shown to stimulate the occurrence of self-healing behavior by introducing ferromagnetic or superparamagnetic particles into the matrix and utilizing the magneto-thermal effect generated by the magnetic field. Cerdan et al. [79] prepared a magnetically initiated self-healing elastomer by incorporating DA bonding and magnetite particles into polymers. The incorporation of DA bonding endows the polymer with self-healing properties, with the magnetite particles acting as the driving particles (Figure 13). A magnet was placed at the damaged section of the elastomer and simultaneously heated to enhance the material’s fluidity. The magnetic field was utilized to induce electromagnetic effects, thereby stimulating the material’s self-healing capabilities, thus facilitating the restoration of its integrity.

**Figure 14:**
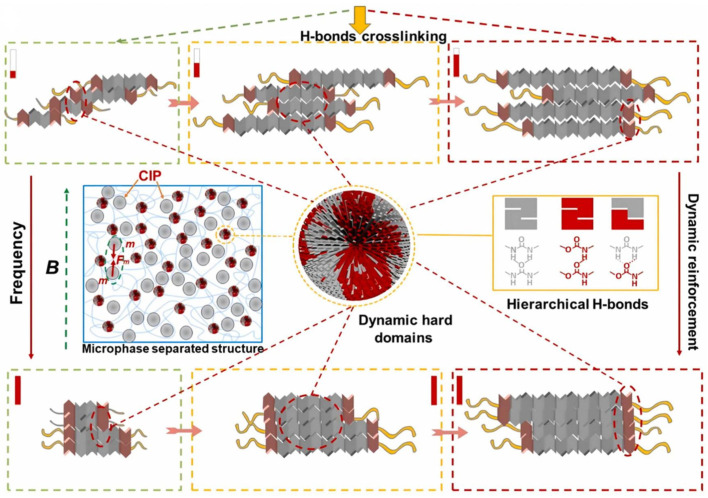
Schematic representation of chemical structures of dynamic hard domains with the stimuli of frequency and magnetic field [80].

Guo et al. [80] developed a novel multifunctional composite material by dispersing carbonyl iron particles in a poly(polyurethane-urea) matrix, creating a self-healing magnetorheological elastomer (SH-MRE) that simultaneously exhibits shear stiffening, magnetorheological effects, and self-healing capabilities. The experimental results demonstrate that the hydrogen bonds within dynamic hard domains and the magnetically induced interactions between carbonyl iron particles are responsible for the material’s excellent multifunctional performance (Figure 14).

## 4. Self-Healing Polyurethanes for Sensor Applications

The advent of the digital era has precipitated the rapid development of intelligent, flexible sensors. Conventional sensors characteristically utilize rigid materials, such as metal or plastic, as the substrate. A notable disadvantage of this approach is that the rigidity of these materials often restricts their flexibility and stretchability, thereby constraining their application and hindering their suitability for diverse requirements within the contemporary field of artificial intelligence. Consequently, flexible sensors that exhibit both good flexibility and stretchability have become a major research focus [81]. Self-healing flexible sensors are of great significance for the new generation of flexible sensors by virtue of the fact that they are able to restore the initial performance of the sensor through external stimulation when damaged [82]. Consequently, polymers with self-healing properties have emerged as a significant substrate material for the development of next-generation flexible sensors.

**Figure 15:**
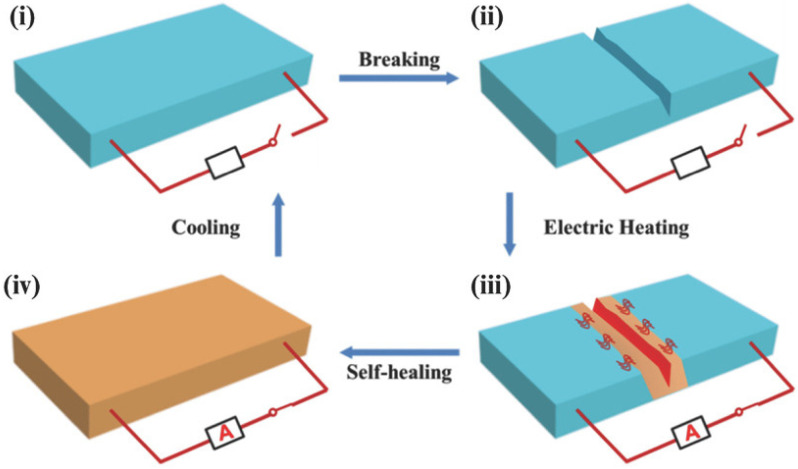
Schematic representation of autonomic electricity-triggered damage reporting and self-healing concept of PUDA/CNTs composite as an electronic device: (**i**) undamaged composite; (**ii**) cut the composite to form a crack; (**iii**) applying an appropriate voltage; and (**iv**) completely healed [83].

Pu et al. [83] prepared a flexible conductive composite with crack diagnostic and self-healing functions, as illustrated in Figure 15. The integration of dynamic adhesives (DAs) within the composite structure was instrumental in facilitating self-healing capabilities. Concurrently, the implementation of carbon nanotube powder onto the polyurethane surface was deliberate, with the objective of enhancing electrical conductivity through a bespoke design. This composite material, designated as flexible conductive polyurethane (PUDA), exhibits the capacity for autonomous healing, thereby demonstrating its innovative nature. It has been demonstrated that the mechanical properties of the conductive polyurethane are significantly improved due to the crosslinking of DA bonds with the polyurethane network. The carbon nanotubes act as a conductive layer in this context but also have excellent photothermal conversion properties and electrothermal effects. This facilitates the conductive polyurethane completing the healing process under both near-infrared light conditions and electrical conditions. Following the application of voltage to the conductive polyurethane, the occurrence of disparate electrothermal effects is evidenced, attributable to the resistance discrepancy between the fractured position and its environs. These effects manifest as discernible color variations in the infrared thermal imaging camera, thereby substantiating the efficacy of crack diagnosis. The high sensitivity of this conductive polyurethane as a sensor demonstrates the potential of polyurethane in the field of flexible sensors.

**Figure 16:**
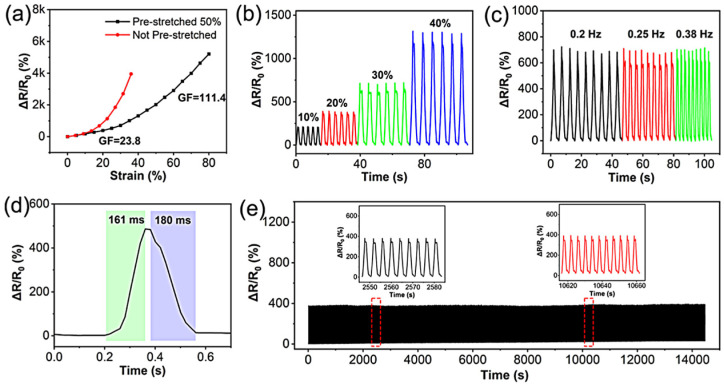
(**a**) GFs of CNT-COOH/PUU_0.5_ strain sensors prepared with and without prestretching. Response tests of the CNT-COOH/PUU_0.5_ strain sensor at (**b**) 10, 20, 30, and 40% strains and (**c**) 0.2, 0.25, and 0.38 Hz. (**d**) Response time and recovery time of the CNT-COOH/PUU_0.5_ strain sensor. (**e**) Response tests of the CNT-COOH/PUU0.5 strain sensor during 3000 stretching−releasing cycles. Insets show the enlarged response curves [84].

Yang et al. [84] prepared a self-healing and degradable polyurethane elastomer (Figure 16). Following the synthesis of a prepolymer, in which polycaprolactone diol (PLC) was utilized as a soft segment and isophorone diisocyanate (IPDI) as a hard segment, the resultant polyurethane elastomer was obtained through a reaction with adipic dihydrazide (ADH). The elastomer’s high healing rate of 92.1% can be attributed to its capacity to form numerous hydrogen bonds during the reaction. In addition, carbon nanotubes were utilized as conductive substances to assemble stretchable strain sensors, which exhibited favorable mechanical properties (16.28 MPa, 660%), stable responsiveness (3000 tensile cycles), high sensitivity (GF = 111.4), and rapid response/relaxation times (161/180 ms). The experimental findings demonstrate the efficacy of strain sensors when assembled on elastomer substrates, thus highlighting their potential for utilization in the monitoring of human motion. This is a crucial aspect for the advancement of sustainable electronic devices.

Sun et al. [85] developed a novel aerogel pore-collapse method to fabricate poly(3,4-ethylenedioxythiophene):poly(styrenesulfonate) (PEDOT:PSS)-incorporated conductive polyurethane composites. The resulting conductive polyurethane elastomers (CPUEs) containing 1.53 wt% PEDOT:PSS demonstrated exceptionally high electrical conductivity (1590 S/m). This innovative processing technique provides new insights for preparing flexible polyurethane elastomers with high conductivity. Gema et al. [86] also utilized PEDOT:PSS to fabricate bioelectronic devices. This approach not only reduces costs and production time but also minimizes environmental pollution while demonstrating enhanced biocompatibility. These advantages further suggest that the technology can be integrated with self-healing polyurethanes to expand applications in bioelectronics and sensor systems.

Self-healing polyurethanes fundamentally address mechanical damage and signal distortion in flexible sensors caused by repeated deformation through dynamic bond reconfiguration and functional self-regeneration mechanisms. This enables seamless coordination between “damage repair and functional maintenance” in devices, providing a key material foundation for practical breakthroughs in high-robustness intelligent sensing systems.

## 5. Conclusions and Prospects

This paper provides a detailed review of the classification, self-healing mechanisms, and research progress of self-healing polyurethane elastomers. Regarding self-healing polyurethane elastomers, they are categorized into two types based on their healing mechanisms: intrinsic self-healing and extrinsic self-healing. Among these, intrinsic self-healing polyurethane can be achieved through the following approaches: self-healing based on reversible non-covalent bonds (including metal-ligand coordination, ionic bonds, and hydrogen bonds) and self-healing based on covalent bonds (including Diels–Alder bonding, reversible acylhydrazone bonds, imine bonds, and disulfide bonds). Depending on the stimulus applied, they can be classified as thermally induced, photoinduced, pH-induced, electrically induced, and magnetically induced self-healing.

In the field of flexible electronics, self-healing polyurethanes have demonstrated immense potential as substrate materials for sensors, significantly enhancing the durability and stability of devices by endowing them with self-repair capabilities. The main trends of self-healing polyurethane will be functional diversification with medical-electronic-environmental integration. However, there are still challenges, including large-scale production with a facile process at low cost, balanced performance with suitable hardness and density, and a standardized testing system.
